# Polyguanine alleviated autoimmune hepatitis through regulation of macrophage receptor with collagenous structure and TLR4‐TRIF‐NF‐κB signalling

**DOI:** 10.1111/jcmm.17599

**Published:** 2022-10-25

**Authors:** Tingchen Cai, Lanman Xu, Dingchao Xia, Lujian Zhu, Yanhan Lin, Sijie Yu, Kailu Zhu, Xiaodong Wang, Chenwei Pan, Yongping Chen, Dazhi Chen

**Affiliations:** ^1^ Department of Infectious Diseases, Zhejiang Provincial Key Laboratory for Accurate Diagnosis and Treatment of Chronic Liver Diseases, Wenzhou Key Laboratory of Hepatology, Hepatology Institute of Wenzhou Medical University The First Affiliated Hospital of Wenzhou Medical University Wenzhou China; ^2^ Department of Infectious Diseases and Liver Diseases, Ningbo Medical Centre Lihuili Hospital, Affiliated Lihuili Hospital of Ningbo University Ningbo Institute of Innovation for Combined Medicine and Engineering Ningbo China; ^3^ Department of Infectious Diseases The Second Affiliated Hospital and Yuying Children's Hospital of Wenzhou Medical University Wenzhou China; ^4^ Department of Clinical Medicine Hangzhou Medical College Hangzhou China

**Keywords:** autoimmune hepatitis, inflammation, macrophage receptor with collagenous structure, polyguanine

## Abstract

Autoimmune hepatitis (AIH) is a progressive and chronic inflammatory disease in the liver. MARCO is a surface receptor of macrophage involving in tissue inflammation and immune disorders. Moreover, polyguanine (PolyG) is considered to bind to macrophage receptor with collagenous structure (MARCO). However, the role of MARCO and PolyG in the development and treatment of AIH still remains unclear. Therefore, this study explores the expression of MARCO and therapeutic activity of PolyG in both S100‐induced AIH in mouse and Lipopolysaccharide (LPS)‐treated macrophage (RAW264.7 cells). Moreover, there were significant increases in inflammatory factors and MARCO, as well as decrease in I‐kappa‐B‐alpha (Ik‐B) in the liver of AIH mice and LPS‐induced cells. However, PolyG treatment significantly reversed the elevation of inflammatory cytokins, MARCO and reduction of Ik‐B. In addition, PolyG treatment could downregulate the expression of Toll‐like receptor 4 (TLR4) and TIR‐domain‐containing adaptor inducing interferon‐β (TRIF), decrease macrophage M1 polarization and increase macrophage M2 polarization. When hepatocytes were co‐cultured with different treatment of macrophages, similar expression profile of inflammatory cytokines was observed in hepatocytes. The research revealed that MARCO expression was elevated in AIH mice. PolyG treatment and inhibition of MARCO significantly reduced inflammatory cytokines expression in the liver as well as hepatocytes and macrophages. Therefore, MARCO could be a target for the treatment of AIH.

## 1 INTRODUCTION

Autoimmune hepatitis (AIH) is a progressive chronic disease of the liver with unknown causes, which features high serum levels of alanine aminotransferase (ALT), aspartate aminotransferase (AST), immunoglobulin G (IgG) and presence of autoantibodies.^1^, ^2^, ^3^ AIH can occur on both gender of different ages and ethnics around the world.^4^ AIH can be presented as asymptomatic or in various forms of conditions such as acute or chronic hepatitis, cirrhosis and end‐stage liver diseases.^5^ The mechanisms of AIH are complex and uncertain, and AIH can progress to severe liver disease. Therefore, there is an urgent need to explore the pathogenesis of AIH and search for potential and effective therapeutic targets.^6^

Macrophage receptor with collagenous structure (MARCO) is a class A scavenger receptor (SR) found on particular subsets of macrophages and mainly located in the spleen, lymph node and the liver.^7^ As SR, MARCO is able to bind bacteria and initiate immunity against bacteria. Although MARCO does not directly cause an inflammatory response, it can help other receptors interact with the pathogen‐associated molecular patterns (PAMPs) to initiate inflammation. AIH involves in activation and interaction of macrophages and lymphocytes leading to a series of inflammatory reactions, hepatocyte apoptosis and even liver failure.^8^ Although the role of MARCO in AIH is still unclear, MARCO participates in part development of hepatocellular carcinoma.^9^

Polyguanine (PolyG) is a stretch of repeated guanine nucleotides, which is synthesized from GDP by thermus thermophilus.^10^ In previous study, PolyG antagonizes the expression of SR MARCO, and it can attenuate silica‐induced pulmonary fibrosis through inhibition of epithelial‐mesenchymal transition.^11^ Interestingly, MARCO‐mediated uptake can be inhibited by its ligand PolyG, but not by polyribonucleotides (PolyC) and polyuridylic acid (PolyU) used as controls.^12^ And it is reported that the four‐fold structure of PolyG is a key structural element for SR identification.^13^ However, there is a lack of fully understanding of the role of PolyG in liver disease. Therefore, in this study, we research the biological activity of MARCO in AIH and explore therapeutic opportunity of PolyG in AIH.

## 2 MATERIALS AND METHODS

### 2.1 Materials and reagents

Primary antibodies against MARCO (ab239369), TRIF (ab13810), NF‐κB (ab16502), TNF‐α (ab183218), Lamin B (ab133741) and GAPDH (ab181602) as well as the secondary antibody of goat anti‐rabbit were purchased from Abcam. Primary antibody against IκB‐α (4814) was purchased from Cell Signalling Technology. Primary antibody against Toll‐like receptor 4 (TLR4) (AF7017) was purchased from Affinity. PolyG (P4404) and LPS (L2880) was purchased from Sigma.

### 2.2 Establishment of AIH model in experimental murine

C57BL/6 male mice were purchased from the Shanghai Charles River which were on 6–8 weeks of age. Our animals care and experimental procedures (Approval Document No. Wydw2020‐0865) were approved by the Wenzhou Medical University Animal Policy and Welfare Committee. All experiments were performed in accordance with the health guidelines of the National Institutional (Guide for the care and use of laboratory animals). Mice were adapted to the laboratory for about 2 weeks before the experiments.

The mouse model of AIH was generated by intraperitoneal injection of freshly prepared S100 into the mice. Liver antigen S100 is produced by perfusion of the liver with normal saline and prepared as described in our previous publication.^14^ Briefly, the liver was minced and homogenized with cold PBS on ice and subsequently centrifuged at 150 *g* for 10 min. Next, supernatants were further centrifuged an 100,000 *g* for 1 h, and resulting supernatants were called S100,^14^ which were used for further separation by concentrating to 5 ml using an Amicon Ultra‐15 filter (Millipore) and then passing through a 90 cm CL‐6B Sepharose column with the AKTA pure (GE Healthcare). There were three protein peaks collected from the column with the peak 2 was toxic components and the peaks 1 and 3 were safe components as liver antigen. In this experiment, the peak 3 component with the concentration of 0.5–2.0 g/L were used. Moreover, for immunization, the liver S100 antigen was emulsified in an equal volume of complete Freund's adjuvant (Solarbio). There was no death of mice in the course of the experiment. There were two groups of mice with 16 established AIH and 16 normal control mice, among them half of mice in each group were treated with PolyG (Sigma Aldrich) randomly. PolyG (2.5 mg/kg) was dissolved in sterile saline and injected into tail vein on day 14 before mice were sacrificed on day 28. The remaining normal control group was injected with PBS. To evaluate the disease severity, three mice were sacrificed for histology and blood biochemistry assay. During the experiment, 10 mice died.

### 2.3 Liver histopathology examination and examination of serum enzyme

Parts of liver tissues which from 32 mice were fixed in paraffin and 4% paraformaldehyde. The 5‐mm sections were prepared and then used to stain with haematoxylin and eosin and incubate with antibody against MARCO for evaluation of lymphocytic infiltration, hepatocyte necrosis and the expression of MARCO in liver, respectively.

The serum levels of alanine transaminase (ALT) and aspartate transaminase (AST) were evaluated using an automatic biochemistry analyser (Abbott Laboratories) from the clinical laboratory department of the first affiliated Hospital of Wenzhou Medical University.

### 2.4 Isolation of hepatic macrophage and flow cytometry analysis

Following the protocol of Dr. Li with collagenase IV digestion, gradient centrifugation and selective adhesion,^15^ macrophages were isolated from normal liver tissue. To distinguish M1 and M2 polarization transitions of hepatic macrophages after PolyG treatments, F4/80, CD11B and CD86 were chosen to label hepatic macrophages M1 phenotype while F4/80, CD11B and CD206 were used to label hepatic macrophages M2 phenotype. PE‐conjugated anti‐Mouse F4/80 (Multi Sciences), FITC‐conjugated anti‐Mouse Human/Mouse CD11B (Multi Sciences), APC‐conjugated anti‐Mouse CD86 (Multi Sciences) and PE‐Cy7‐conjugated anti‐Mouse CD206 (ebioscience) were used to evaluate macrophage subsets. Flow cytometry data were analysed using CytExpert software.

### 2.5 Cell culture and transfection

RAW264.7 cells were maintained in low‐glucose Dulbecco's modified Eagle's medium solution (Gibco) with 10% FBS (Gibco), 40 ng/ml dexamethasone (Gibco) and 1% ITS (I3146; Sigma) at 37°C in 5% CO_2_ incubator. AML12 cells were cultured in DMEM/F‐12 (Gibco) with 10% FBS, 1% ITS and 40 ng/ml dexamethasone at 37°C in 5% CO_2_ incubator. Cells were treated with PolyG (1 mg/ml) and LPS (100 ng/ml, L2880) for 24 h after cells were subcultured and grown to 80% confluence. When cells were subcultured and grown to 40%–50% confluence, according to the manufacturer's procedure, cells were transfected with MARCO‐specific siRNA, respectively, with GP‐transfect‐Mate (GenePharma) and non‐specific control short inhibitory RNA (siRNA). Cell culture medium was changed to DMEM for 4–6 h and then in DMEM with 10% FBS for 24–36 h after transfection. The siRNAs specific to MARCO and non‐specific control siRNA were all purchased from GenePharma. Table S1 lists the sequences for non‐specific control siRNA and MARCO specific siRNA.

### 2.6 Cell viability assay with CCK‐8

CCK‐8 (Dojindo) was used to determine the cell viability of control group and treatment groups with different concentrations of PolyG. Cells were seeded in 96‐well plate at a density of 10^4^–10^5^ cells per well and treated with various concentrations of PolyG (0.2, 0.4, 0.6, 0.8, and 1 mg/ml) dissolved in 0.1% DMSO or 0.1% DMSO alone for control. Cells were maintained in a 5% CO_2_ incubator at 37°C for 24 h and then 10 ml of CCK‐8 solution was added into each well of the plate using a repeating pipettor. The plates were further incubated for 2 h, and the absorbance of each well were measured by using a microplate reader at wavelength at 450 nm.

### 2.7 Western blot analysis

Cellular lysates were prepared from liver tissue, RAW264.7 and AML12 cells, respectively, by homogenizing tissues or cells in the lysis buffer (AR0101/0103; Boster Biological Technology Co. Ltd.). According to the manufacturer's protocol, the Pierce BCA protein assay kit (P0010; Beyotime) was used to detect protein concentration. After heat denaturation at 100°C for 10 min, 50 μg of liver tissue and cell lysates were into SDS‐PAGE and separated by electrophoresis, and then proteins were transferred to polyvinylidene fluoride membranes (Millipore). The membranes were first blocked with 5% skim milk and then incubated with the primary antibodies against MARCO, TLR4, TRIF, TNF‐a, IkB‐a, NF‐kB, Lamin B and GADPH, respectively at 4°C overnight or at room temperature for 2 h followed by incubation of the secondary antibodies conjugated with horseradish peroxidase at room temperature for 1–1.5 h. The bands were displayed with enhanced chemiluminescence reagent (Bio‐Rad). Then the protein band density was analysed by Image J or Image Lab analysis software and normalized to their respective controls.

### 2.8 RNA isolation and qRT‐PCR analysis

Total RNA was isolated from frozen liver tissue, RAW264.7 cells and AML12 cells using TRIzol reagent (Life Technologies) with the commercialized protocol. RNA levels were used to synthesize cDNA by the Prime ScriptTM RT reagent kit (Takara). Quantitative real‐time PCR was performed to determine messenger RNA levels of MARCO, TNF‐a, NF‐kB, IkB‐a, IL‐1b and IL‐6 using TB Green Premix Ex TaqTM II (Takara) in ABI7500 Fast real‐time PCR system (Applied Biosystems). The 2−△CT method was used to normalize the expression of a single gene to the expression of GAPDH, and the 2−△△CT method was used to calculate the amount of messenger RNA relative to the reference gene. Table S2 lists the primers' sequences.

### 2.9 Co‐culture of RAW264.7 and AML12

RAW264.7 cells and AML12 cells were co‐cultured to simulate the microenvironment between macrophages and hepatocytes. Assay was carried out in a 24‐well plate with a permeable cross‐hole insert (aperture 0.4 mm; Corning). AML12 cells were cultured in the lower chambeat a density of 4 × 10^5^ cells/cm^2^, RAW264.7 cells (4 × 10^5^ cells/cm^2^) were cultured in the upper chamber. After RAW264.7 cells were treated with DMSO (control), LPS, PolyG and LPS + PolyG, respectively, RAW264.7 cells were then co‐cultured with AML12 cells at 37°C in a 5% CO_2_ incubator. After 24 h co‐culture, AML12 cells were collected for RT‐qPCR and Western blotting analyses.

### 2.10 Statistical analysis

The data were analysed By GraphPad Prism 8 software. All experiments were repeated at least three times. Student's *t*‐test was evaluated the statistical significance of two groups. One‐way anova or two‐way anova with Bonferroni test or Fisher's LSD test was evaluated the statistical significance between multiple groups. Data were shown as mean ± SEM. *p* < 0.05 was considered statistically significant difference.

## 3 RESULTS

### 3.1 Viability of cells

**FIGURE 1:**
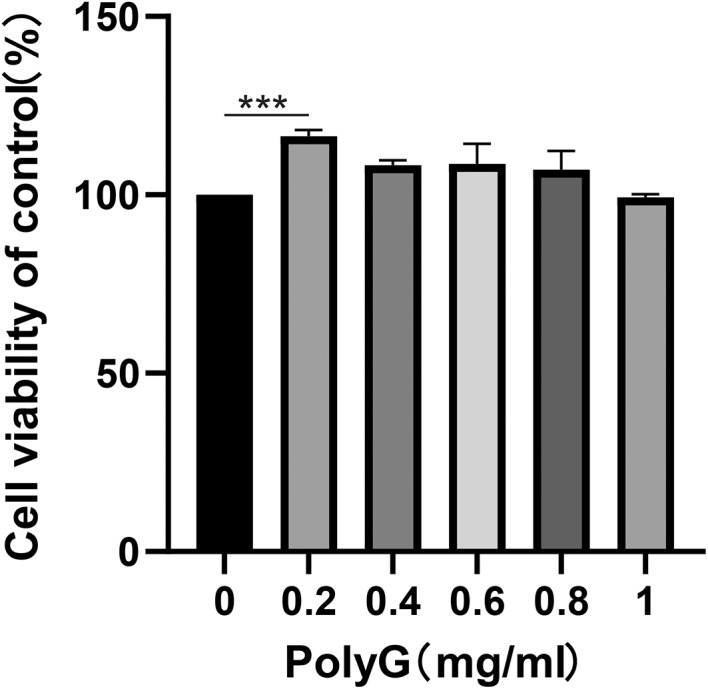
Effects of PolyG on RAW264.7 cell viability. RAW264.7 cells were treated with different concentrations of PolyG (0, 0.2, 0.4, 0.6, 0.8, 1 mg/ml), and cell viability was assessed by CCK‐8 assay. Data are presented as mean ± SEM and *** indicates *p* < 0.001 compared to control.

In order to study the role of PolyG in RAW264.7 cells, optimal concentration of PolyG was determined in RAW264.7 cells. As shown in Figure 1, when RAW264.7 cells were treated with different PolyG concentrations from 0.2, 0.4, 0.6, 0.8 to 1 mg/ml, the cell viability was almost 100% at PolyG concentrations in the range of 0.4–1 mg/ml. The PolyG concentration at 0.2 mg/ml had obvious effects on cell viability (*p* < 0.01). Based on the preliminary results, the experimental concentrations of PolyG were finally determined to be 1 mg/ml.

### 3.2 Establishment of murine AIH model and regulation of SR MARCO in this model

**FIGURE 2:**
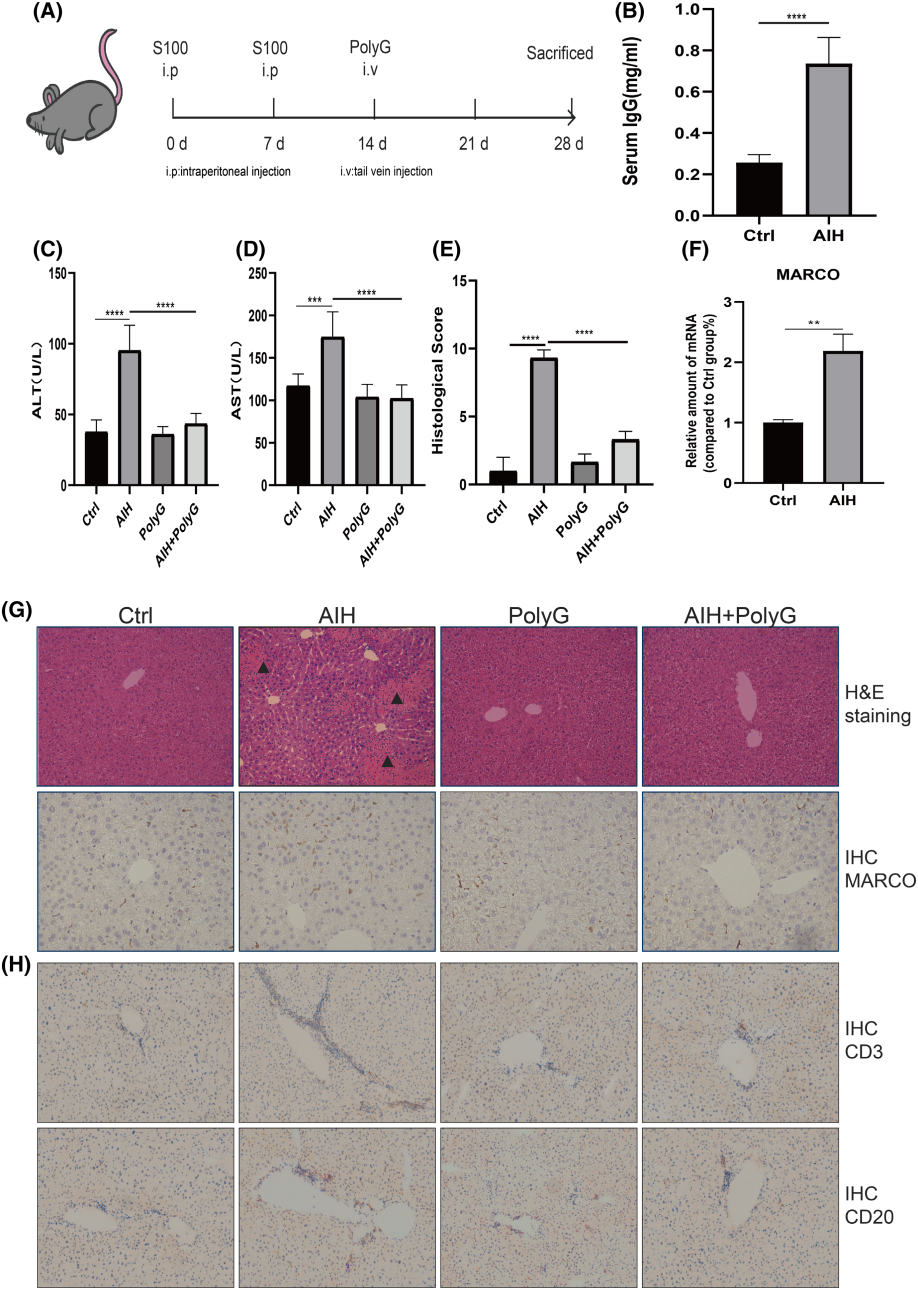
Establishment of murine autoimmune hepatitis (AIH) model and PolyG alleviates the liver inflammation in AIH mice. Panel A shows the protocol to establish the AIH in mice and injection of PolyG. Panel B displays the levels of immunoglobulin G in two group. Panels C and D represent the levels of serum alanine aminotransferase (ALT), aspartate aminotransferase (AST) in each group. Panel E shows histology scores (based on ishak inflammation score). Panel F shows the mRNA levels of macrophage receptor with collagenous structure (MARCO) in the liver tissues from control (Ctrl) and AIH group. Panel G shows the representative pictures of haematoxylin and eosin staining (upper panel) and MARCO immunohistologic staining (lower panel). Panel H displays CD3 (upper panel) and CD20 (lower panel) immunohistologic staining (magnification, ×100). Black triangle highlights the lymphocytic infiltration (magnification, ×200). Data are presented as mean ± SEM from 8 mice. ** indicates *p* < 0.01, *** indicates *p* < 0.001, and **** indicates *p* < 0.0001.

Figure 2A shows the protocol to establish the mouse model of AIH. S100 antigen was injected into the mouse's intraperitoneal cavity on day 0 and day 7, while PolyG was injected into mouse through the tail vein on day 14. Mice were sacrificed on day 28. Liver damage and hepatitis were evaluated by determination of ALT and AST as well as histological staining with haematoxylin and eosin. Moreover, the expression of MARCO was determined by RT‐PCR and immunohistologic staining with antibody against MARCO as shown in Figure 2F,G. Compared to the Control group and the CFA group, there were significant increases in ALT (Figure 2C, Figure S1A) and AST (Figure 2D, Figure S1A) in AIH mice which mean the success of establishment of murine model of AIH. Moreover, PolyG treatment significantly reduced ALT and AST levels. Histological staining with haematoxylin and eosin also showed liver inflammation with lymphocytic infiltration in the portal area (Figure 2E, Figure S1B). PolyG treatment obviously reduced levels of liver inflammation in AIH mice with no alternation of the liver histology with PolyG in normal mice (Figure 2G,H, Figure S2B,C). In addition, MARCO mRNA levels are elevated (Figure 2F) in the liver tissue of AIH mice. And there was increased MARCO staining in the livers of AIH mice, while PolyG treatment reduced the staining of MARCO in AIH mice (Figure 2G, Figure S2A). Important increases in inflammation factors levels were observed in the CFA group compared to the Control group. Inflammation factors levels increased significantly in AIH group relative to values of the Control group and the CFA group (Figure S1D).

### 3.3 PolyG attenuated inflammation of AIH in mice

**FIGURE 3:**
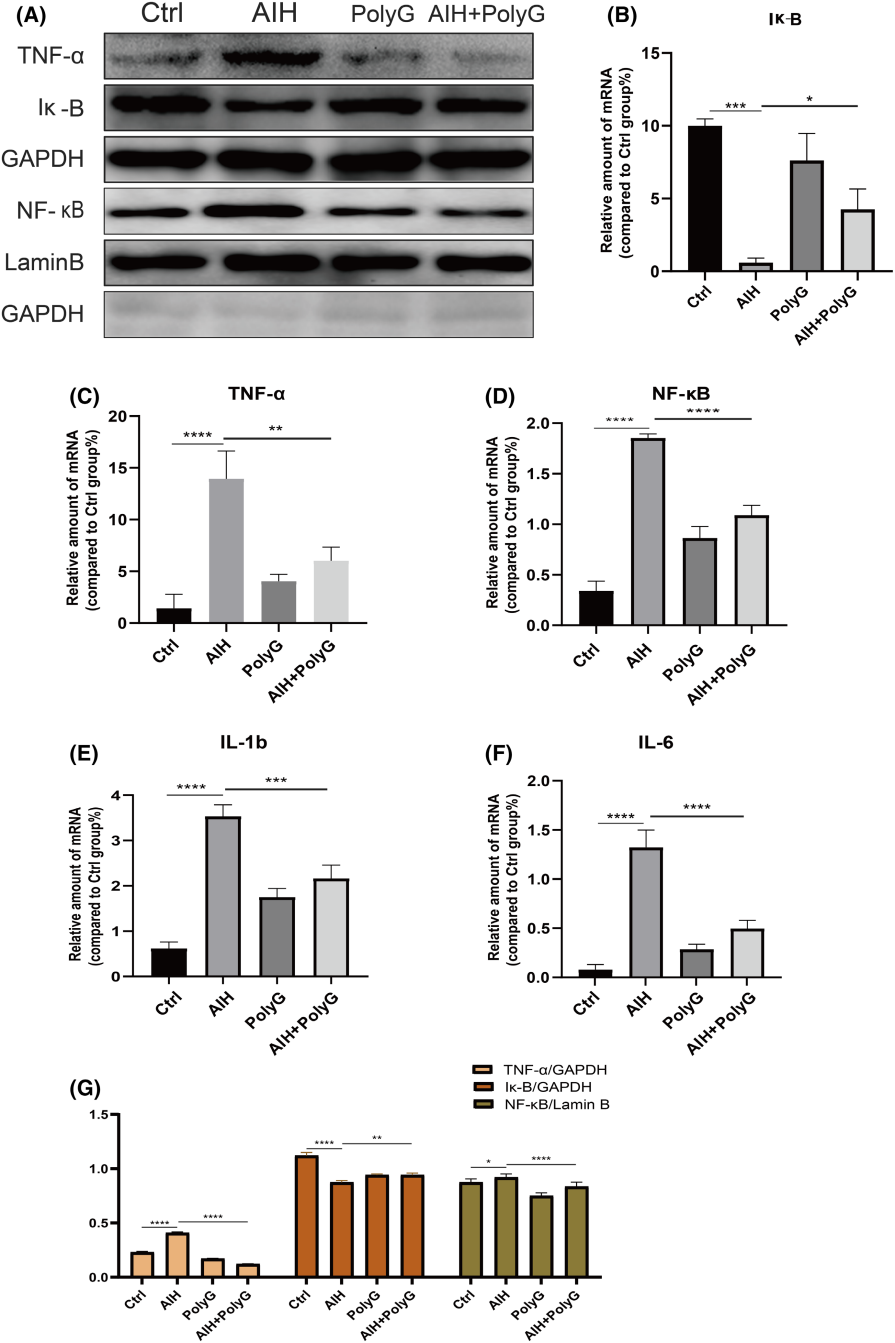
The protein expression and mRNA levels of inflammatory factors in the liver of autoimmune hepatitis (AIH) mice. Panel A displays the representative Western blot for TNF‐a, Ik‐B and NF‐kB. Panels B to F show the mRNA levels of inflammation cytokines in the liver in each group. Panel G represents histogram of proteins for TNF‐a, Ik‐B and NF‐kB. GAPDH and Lamin B were used as loading control. Data are presented as mean ± SEM from 8 mice. ** indicates *p* < 0.01, *** indicates *p* < 0.001, and **** indicates *p* < 0.0001.

We use different methods to deal with mice. The expression of inflammatory cytokines and intracellular signalling molecules was examined in the AIH liver without and with treatment of PolyG. As shown in Figure 3A, TNF‐a, Ik‐B and NF‐kB proteins were estimated by Western blot, and mRNA levels of TNF‐a, Ik‐B, NF‐kB, IL‐1b and IL‐6 were examined by RT‐PCR. The protein levels of TNF‐a and NF‐kB were significantly increased in the liver of AIH mice while PolyG treatment significantly reduced the levels. However, the protein level Ik‐B was significantly reduced in the liver of AIH mice while PolyG treatment significantly elevated the level (Figure 3A,G). The mRNA levels of TNF‐a, Ik‐B and NF‐kB in the livers of AIH mice treated with and/or without PolyG showed similar changes as their proteins (Figure 3B–D). Moreover, the mRNA levels of IL‐1b and IL‐6 exhibited similar changes as TNF‐a as increase in the liver of AIH and decrease in the liver of AIH with PolyG treatment (Figure 3E,F).

### 3.4 PolyG alleviates LPS‐mediated release of pro‐inflammatory cytokines in RAW264.7 cells

**FIGURE 4:**
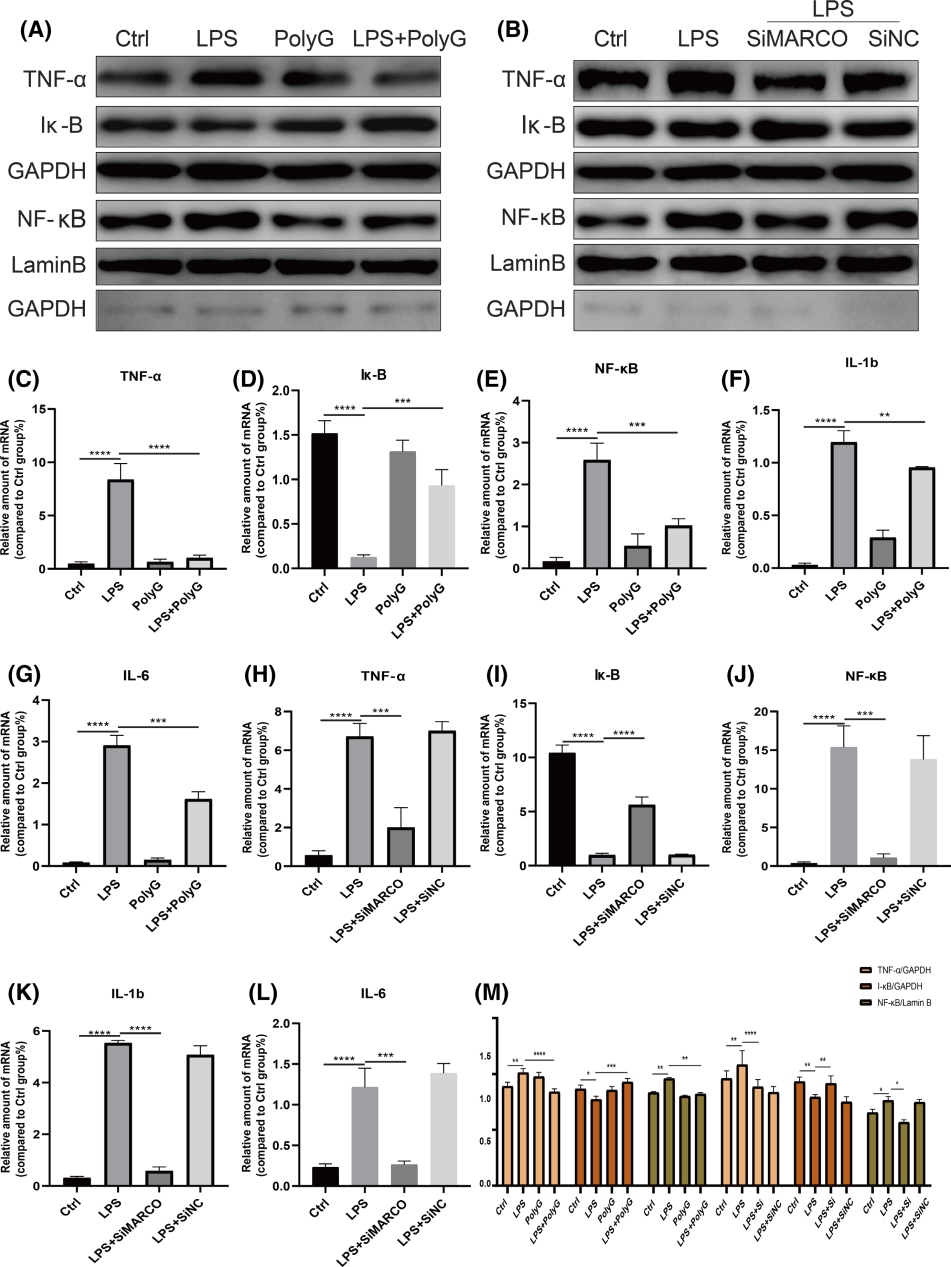
PolyG attenuates inflammatory cytokine expression in LPS‐induced RAW264.7 cells. Panel A displays the typical Western blot for TNF‐a, Ik‐B and NF‐kB from RAW264.7 cells treated with LPS plus or minus PolyG. Panel B shows the representative Western blot from RAW264.7 cells treated with LPS and specific inhibitory siRNA of macrophage receptor with collagenous structure (MARCO). GAPDH and Lamin B were used as loading control. Panel C to G shows the mRNA levels of inflammatory cytokines from RAW264.7 cells treated with LPS plus or minus PolyG. Panels H to L represent the mRNA levels of inflammatory factors from RAW264.7 cells treated with LPS and specific inhibitory siRNA of MARCO. Panel M displays histogram of proteins for TNF‐a, Ik‐B and NF‐kB in each group. ** indicates *p* < 0.01, *** indicates *p* < 0.001, and **** indicates *p* < 0.0001.

Macrophages play an important role in the inflammatory response. They are sensitive to LPS, which can induce the release of pro‐inflammatory cytokines through the NF‐κB pathway. In order to better understand the mechanism of MARCO in liver inflammation, RAW264.7 cells were employed and stimulated with LPS, and pro‐inflammatory cytokines expression was examined. As shown in Figure 4A, after the stimulation of LPS, the expression of inflammatory cytokine TNF‐α and intracellular signalling molecule NF‐κB was increased and the expression of cellular Iκ‐B was decreased (Figure 4A,M). Moreover, mRNA levels of these proteins showed the same trend (Figure 4C–E). Correspondingly, LPS‐treated RAW264.7 cells also showed up‐regulated expression of the IL‐1β and IL‐6 mRNA (Figure 4F,G). Furthermore, PolyG treatment significantly reversed the LPS‐induced expression of these inflammatory cytokines and NF‐kB signalling molecules in RAW264.7 cells (Figure 4A). In addition, when the non‐specific and specific inhibitory siRNA of MARCO was incubated with LPS‐treated RAW264.7 cells, the specific inhibition of MARCO significantly reduced LPS‐induced expression of TNF‐a, NF‐kB, IL‐1b and IL‐6 proteins while increased the level of LPS‐reduced Ik‐B protein (Figure 4B,M) while the mRNA levels of these proteins showed similar changes as their proteins (Figure 4H–L).

### 3.5 PolyG inhibits the polarization of M1 macrophages and promotes the polarization of M2 macrophages in AIH

**FIGURE 5:**
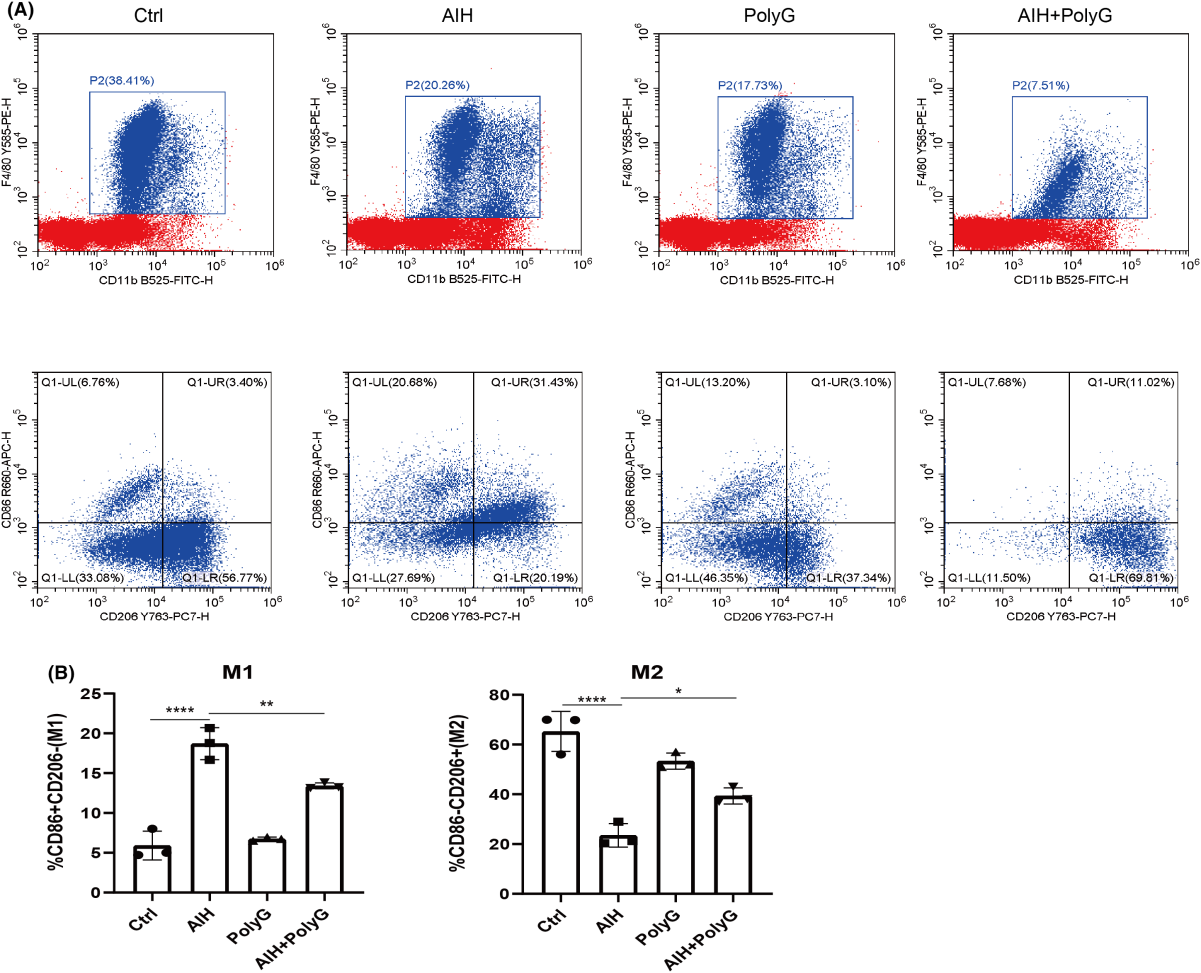
The effect of PolyG on the polarization of liver macrophages in autoimmune hepatitis (AIH) mice. The panel A shows F4/80 + CD11B + CD86 + M1 macrophages, and the panel B displays F4/80 + CD11B + CD206 + M2 macrophages from control and different groups of AIH mice. The panel C shows the percentage of M1 and M2. Data are presented as mean ± SEM from three mice. * indicates *p* < 0.1, ** indicates *p* < 0.01 and **** indicates *p* < 0.0001.

In the AIH model murine, flow cytometry showed that the proportion of liver M1 macrophages increased, and the proportion of M2 macrophages decreased in the liver (Figure 5A). Treatment of PolyG could reduce the proportion of M1 macrophages and increase the proportion of M2 macrophages in the liver of AIH mice, indicating that PolyG may inhibit the polarization of M0 cells to M1 macrophages and promote the polarization of M0 cells to the M2 phenotype macrophages.

### 3.6 PolyG inhibition of MARCO and TLR4/TRIF signalling to alleviate inflammatory cytokine expression in AIH mice and RAW264.7 cells stimulated by LPS

**FIGURE 6:**
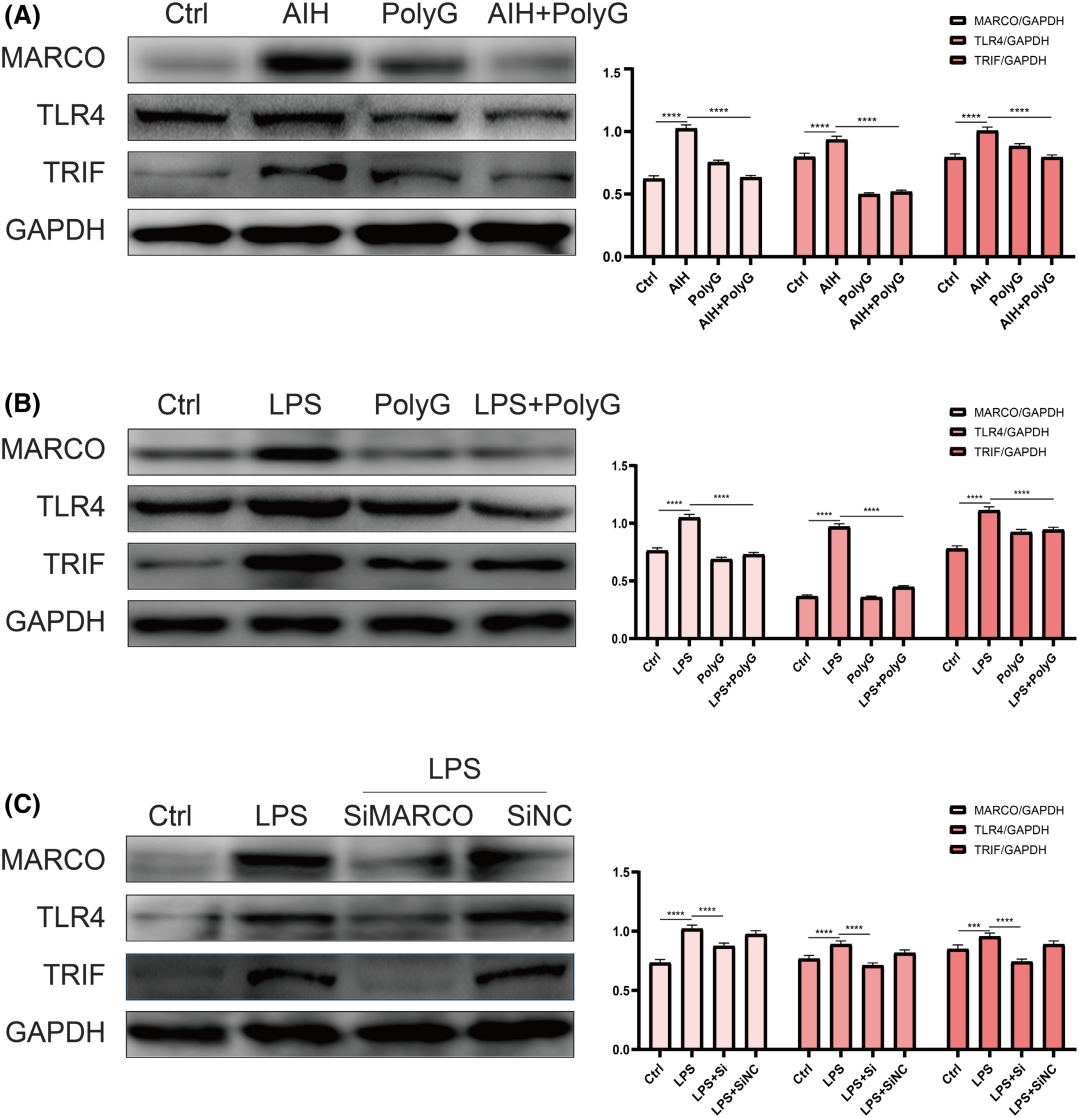
The protein level of macrophage receptor with collagenous structure (MARCO) and TLR4/ TRIF signalling in autoimmune hepatitis (AIH) mice and LPS treated RAW264.7 cells. Panel A shows the representative Western blot and histogram of proteins for MARCO, TLR4, TRIF and NF‐κB in AIH mice treated with and without PolyG. Panel B displays the typical Western blot and histogram of proteins for MARCO, TLR4, TRIF and NF‐κB from RAW264.7 cells treated with LPS plus or minus PolyG. Panel C displays the typical Western blot and histogram of proteins for MARCO, TLR4, TRIF and NF‐κB from RAW264.7 cells treated with LPS and specific inhibitory siRNA of MARCO. GAPDH was used as loading control. Data are presented as mean ± SEM from eight mice. ** indicates *p* < 0.01, *** indicates *p* < 0.001, and **** indicates *p* < 0.0001.

In order to understand the regulation of MARCO expression in AIH mice and LPS‐stimulated RAW264.7 cells. The expression of MARCO and TLR4/TRIF signalling was explored. As shown in Figure 6A, the expression of MARCO, TLR4 and TRIF was significantly increased in the liver of AIH mice. However, PolyG treatment significantly reduced the protein levels of MARCO, TLR4 and TRIF in the liver of AIH mice. Moreover, LPS significantly stimulated the expression of MARCO, TLR4 and TRIF in RAW264.7 cells and PolyG treatment reduced LPS‐induced protein levels of these factors (Figure 6B). Furthermore, LPS induced the expression of MARCO, TLR4 and TRIF in RAW264.7 cells was also reversed by specific inhibitory siRNA of MARCO, indicating that MARCO also mediated the regulation of TLR4/TRIF signalling (Figure 6C). In addition, as shown in Figure 6C, LPS significantly increased the amount of MARCO in RAW264.7 cells.

### 3.7 Co‐culture of RAW264.7 and AML12 can alleviate the inflammatory level of AML12 induced by LPS

**FIGURE 7:**
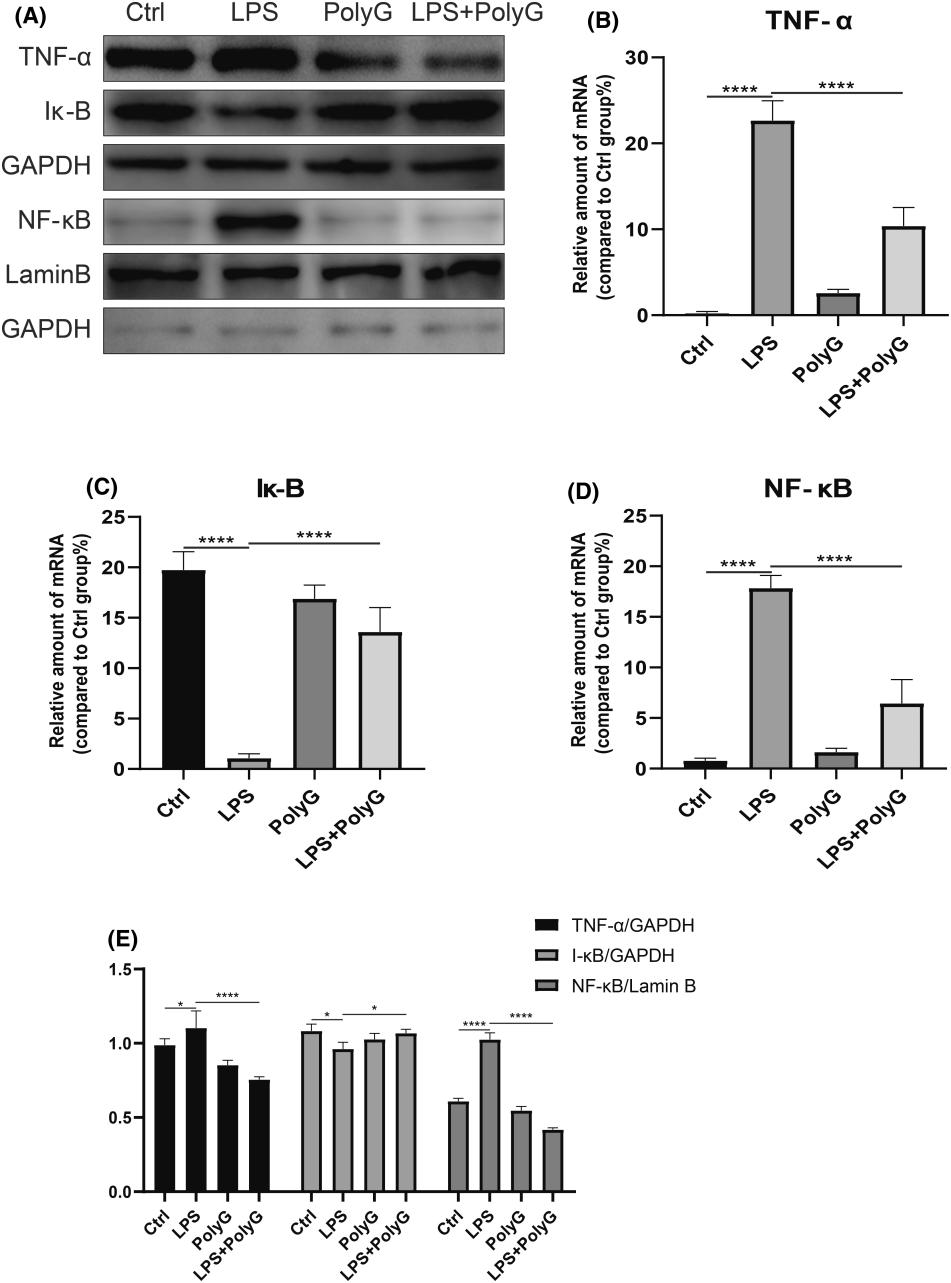
Expression of TNF‐a, Ik‐B and NF‐kB in hepatocytes (AML12 cells) after co‐culture with macrophages (RAW264.7 cells). Panel A displays the typical Western blot for TNF‐a, Ik‐B and NF‐kB from AML12 after co‐culture with RAW264.7 cells treated with LPS and PolyG. Panels B to D show the mRNA levels of inflammation cytokines from AML12 after co‐culture with RAW264.7 cells treated with LPS and PolyG. Panel E represents histogram of proteins for TNF‐a, Ik‐B and NF‐kB from the panel A. GAPDH and Lamin B were used as loading control. * indicates *p* < 0.1, ** and **** indicates *p* < 0.0001.

In order to understand the interaction of macrophage and hepatocytes in the liver, both AML12 cells (hepatocytes) and RAW264.7 cells (macrophage) were employed in a co‐culture environment. The LPS‐induced RAW264.7 cells were incubated with AML12 cells. The expression of TNF‐a, Ik‐B and NF‐kB was evaluated in AML12 cells. As shown in Figure 7, both protein and mRNA levels of TNF‐a and NF‐kB were significantly increased in AML12 cells after co‐cultured with LPS stimulated RAW264.7 cells, while they were reduced when co‐incubation with LPS and PolyG treated RAW264.7 cells. The protein and mRNA levels of Ik‐B showed opposite direction in AML12 cells when they are co‐incubated either LPS stimulated or LPS plus PolyG treated RAW264.7 cells Figure 7A,C.

## 4 DISCCUSION

Autoimmune hepatitis is a progressive and chronic inflammatory liver disease characterized by specific liver histological features such as borderline hepatitis, obvious infiltration of lymphocytes and plasma cells, as well as present of autoantibodies.^2^ Although immunosuppressive therapy can improve the long‐term prognosis of AIH patients, corticosteroids or combination of low‐dose corticosteroids and azathioprine still present significant adverse effects for patients with AIH.^16^ For example, long‐term immunosuppressive therapy may increase body weight of patients as well as cause low‐traumatic fractures, diabetes and vascular diseases.^17^ Therefore, treatment of AIH patients still is a significant challenge. However, recent researches have shown some lights in understanding and treatment of AIH. For example, Tu et al. demonstrated that miRNA‐143 can help reduce AIH‐induced liver inflammation and fibrosis,^18^ and Xu et al. found that Vialinin A inhibits USP4 and affects the Rheb/mTOR pathway to slow down the progression of fibrosis in AIH.^19^

Consistent with previous experiments, earlier experimental studies showed that hepatocyte injury induced by intraperitoneal immunization with the CFA antigen had a marked increase in histological hepatitis and transaminase levels, whereas only minor changes were observed with CFA injection itself.^14^, ^20^

In the current study, we further revealed a role of MARCO in AIH with the treatment of PolyG, which can bind to MARCO. MARCO is a characteristic SR in macrophages and shaped as a coiled homotrimer. It is also a non‐opsonizing phagocytic receptor with good characteristics on myeloid cells and has the ability to bind certain polyanions. Since MARCO can perceive and eliminate pathogens by recognizing PAMPs,^7^, ^14^, ^21^, ^22^ liver macrophages play an important role in the inflammatory response of the liver^23^, ^24^ through release a variety of inflammatory mediators (TNF‐α, IL‐1β and IL‐6).^25^ Moreover, PolyG can inhibit ERS‐related apoptosis and EMT by antagonizing the macrophage receptor MARCO in the lungs and alleviate pulmonary fibrosis caused by silicosis.^11^ In addition, compared with normal liver tissue, the expression of Marco was significantly reduced in liver tissue of liver cirrhosis and liver cancer, suggesting that Marco may play a protective role in liver cirrhosis and liver cancer.^26^, ^27^

Previous studies indicated that MARCO is expressed in subpopulations of macrophages located in the peritoneum, marginal zone of the spleen and the myelin cord.^7^ Moreover, MARCO is also expressed in other tissues such as macrophages in the liver. Although individual studies demonstrated by RNA sequencing that MARCO had a tolerogenic function about inflammation in the human liver,^28^ the function of MARCO and its effect on immunity in the liver remain to be investigated. The current investigation explored a role of MARCO in AIH and found that PolyG, which can bind to MARCO, can significantly reduce elevated ALT and AST levels in AIH mice. There is also reduced levels of lymphocyte and plasma cell infiltration in the liver of AIH mice. In addition, PolyG decreased elevated levels of inflammatory factors in the liver of AIH mice. Furthermore, in vitro study by employing LPS as inflammatory stimulator showed PolyG could also inhibited LPS induced inflammatory cytokines expression. In addition, we also observed that MARCO is highly expressed in M1‐type macrophages at both mRNA and protein levels.^29^ M1 macrophages usually trigger intracellular cell death mechanisms by producing pro‐inflammatory cytokines and chemokines, thereby destroying adjacent tissues. M2 macrophages promote inflammation subsidence by releasing anti‐inflammatory cytokines and repair adjacent tissues.^30^ The current investigation also found that treatment of PolyG could decrease M1 type macrophages and increase M2 type macrophages, thereby promotion of M2 type macrophages could relieve the inflammation in the liver of AIH mice. To simulate the microenvironment of macrophages and hepatocytes in the liver, we established a co‐culture system of RAW264.7 cells and AML12 cells. In co‐culture experiment, after PolyG treatment, the expression of inflammatory cytokines in AML12 cells was decreased. This result is consistent to the observation that PolyG treatment decreased M1 type macrophages and increased M2 type macrophages. Since M2 macrophages is anti‐inflammation, the shift of M1/M2 macrophages could contribute to the reduction of inflammatory cytokines in hepatocytes.

TLR4 is highly expressed in monocytes and plays an important role in pattern recognition. Moreover, TLR4 is also found on Kupffer cells, hepatocytes and mesenchymal cells.^31^, ^32^ As an important macrophage conservative pattern recognition receptor, TLR4 can trigger the TRIF signalling pathway to activate downstream NF‐kB, thereby promoting inflammation.^32^, ^33^ TLR4/TRIF/NF‐κB is a classic signalling cascade, which mediated chronic inflammation and lipid disorders in non‐alcoholic fatty liver.^34^, ^35^, ^36^ Furthermore, a report indicated that early blocking of TLR2/4 ligand can effectively reduce liver damage caused by AIH.^37^ Therefore, we explored the relationship between MARCO and TLR4/TRIF/NF‐κB pathway in AIH. After antagonizing MARCO in AIH, the protein expression of TLR4 was decreased, which further affects the downstream TRIF/NF‐κB pathway and alleviates the progression of the disease. In addition, the SR can be the co‐receptor of TLR4 and HMGB1 (high mobility group protein 1). HMGB1 binds to the class A SR, which is necessary for the effective activation of TLR4.^38^ This could be the reason that PolyG binds to MARCO, which affects the activation of TLR4 and the downstream NF‐κB pathway. The current investigation shows that inhibition of MARCO not only affects the activation of TLR4 but also reduces the expression of TLR4 protein. However, the specific mechanism still needs further investigation.

Our study has some important limitations that are worth mentioning. First of all, S100 and complete Freund's adjuvant were regarded as a whole model to establish a mouse AIH model when we modelled the group, and no CFA alone as a control. Secondly, our experiments were basic experiments included in vivo experiments (C57LB/6 mice) and in vitro experiments (RAW264.7 cells and AML12 cells), but we did not further analyse the changes in healthy people and AIH patients. These need to be explored in the next experiments.

In summary, the research reveals that there was an increased expression of MARCO in the liver of AIH mice, and the ligand of MARCO‐PolyG was able to attenuate inflammatory factors expression in both the liver of AIH and the LPS‐induced macrophages (RAW264.7 cells). Moreover, PolyG could regulate the polarization of M1/M2 macrophages, which results in the regulation of interaction between macrophages and hepatocytes. Furthermore, PolyG could also affect TLR4/TRIF/NF‐κB pathway. However, the expression of MARCO in the liver of patients with AIH and the clinical application of PolyG for the treatment of AIH still need to be further studied.

## AUTHOR CONTRIBUTIONS

**Tingchen Cai:** Conceptualization (lead); data curation (lead); formal analysis (lead); methodology (lead); writing – original draft (lead). **Lanman Xu:** Writing – review and editing (lead). **Dingchao Xia:** Conceptualization (equal); methodology (lead). **Lujian Zhu:** Conceptualization (equal); methodology (equal). **Yanhan Lin:** Methodology (equal). **Sijie Yu:** Methodology (equal). **Kailu Zhu:** Methodology (equal). **Xiaodong Wang:** Supervision (lead). **Chenwei Pan:** Supervision (lead). **Yongping Chen:** Conceptualization (lead); methodology (lead); visualization (lead); writing – review and editing (lead). **Dazhi Chen:** Conceptualization (equal); funding acquisition (lead); methodology (equal); writing – original draft (lead).

## FUNDING INFORMATION

This research was supported by Youth Science and Technology Innovation Cultivation Fund of Peking University School of Medicine (BMU2020PYB005), Wenzhou Science and Technology Bureau major scientific and technological innovation to attack health care projects (No. ZY2019008), Zhejiang Provincial Natural Science Foundation of China (No. LD21H030002 and No. LY21H030004), the National Natural Science Foundation of China (No. 81770585 and No. 82070593), Foundation of Zhejiang Provincial Key Laboratory for Accurate Diagnosis and Treatment of Chronic Liver Disease (grant number 2020E10014‐002) and Medicine Health Project of Zhejiang Province, China (grant number 2020KY276).

## CONFLICT OF INTEREST

The authors have no relevant affiliations or financial involvement with any organization or entity with a financial interest in or financial conflict with the subject matter or materials discussed in the manuscript. This includes employment, consultancies, honoraria, stock ownership or options, expert testimony, grants or patents received or pending or royalties.

## Supporting information


Figure S1

Figure S2

Tables S1‐S2

Figure captions

## Data Availability

All data generated or analysed during this study are included in this published article and all data used to support the findings of this study are available from the corresponding author upon request.
