# Supplementary figures and images for: Polyguanine alleviated autoimmune hepatitis through regulation of macrophage receptor with collagenous structure and TLR4‐TRIF‐NF‐κB signalling
Source: J Cell Mol Med. 2022 Oct 25;26(22):5690–701. doi: 10.1111/jcmm.17599 (PMC9667514; doi:10.1111/jcmm.17599)

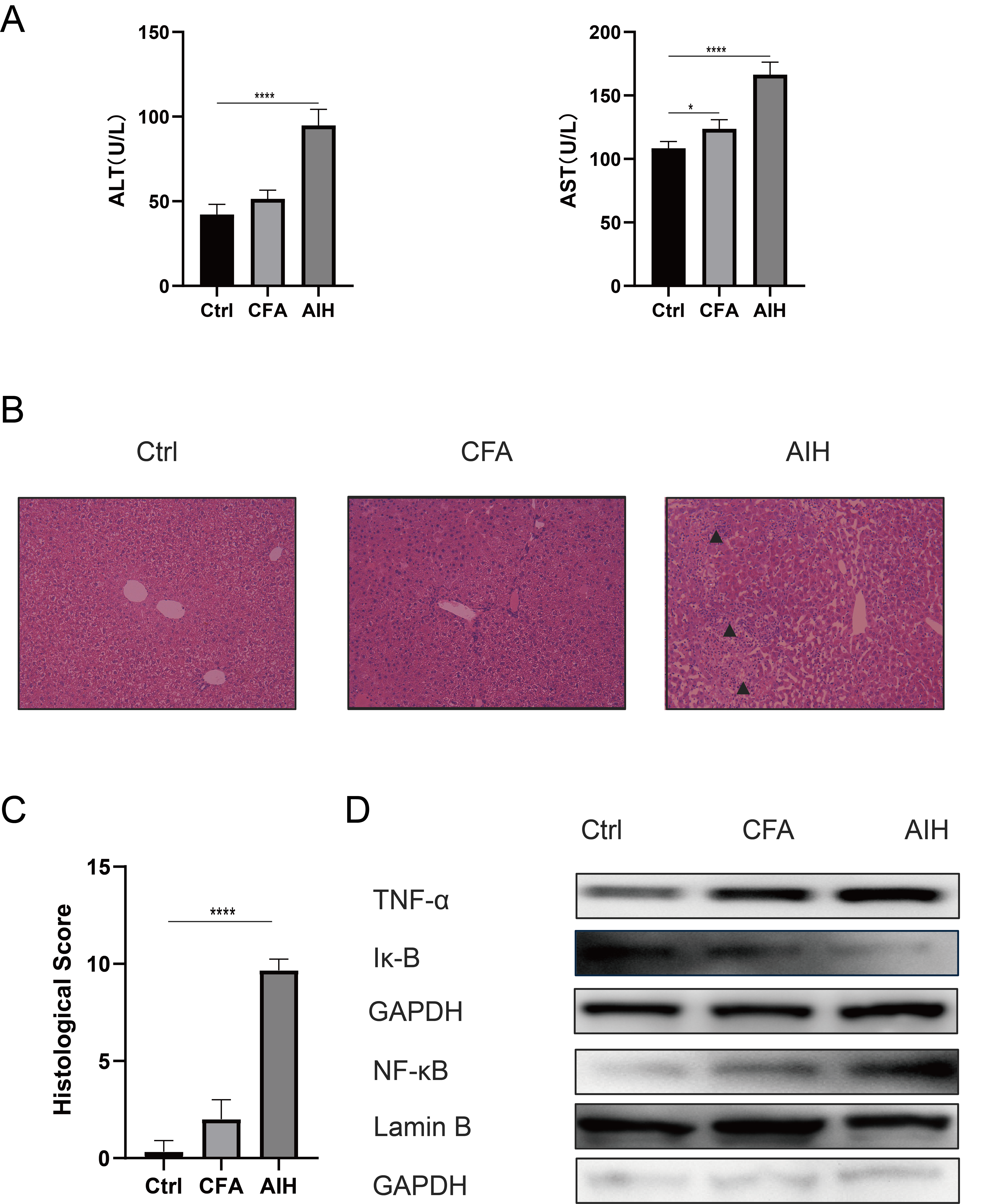

Supplement: Supplementary file 1 — Figure S1 [file JCMM-26-5690-s001.png]

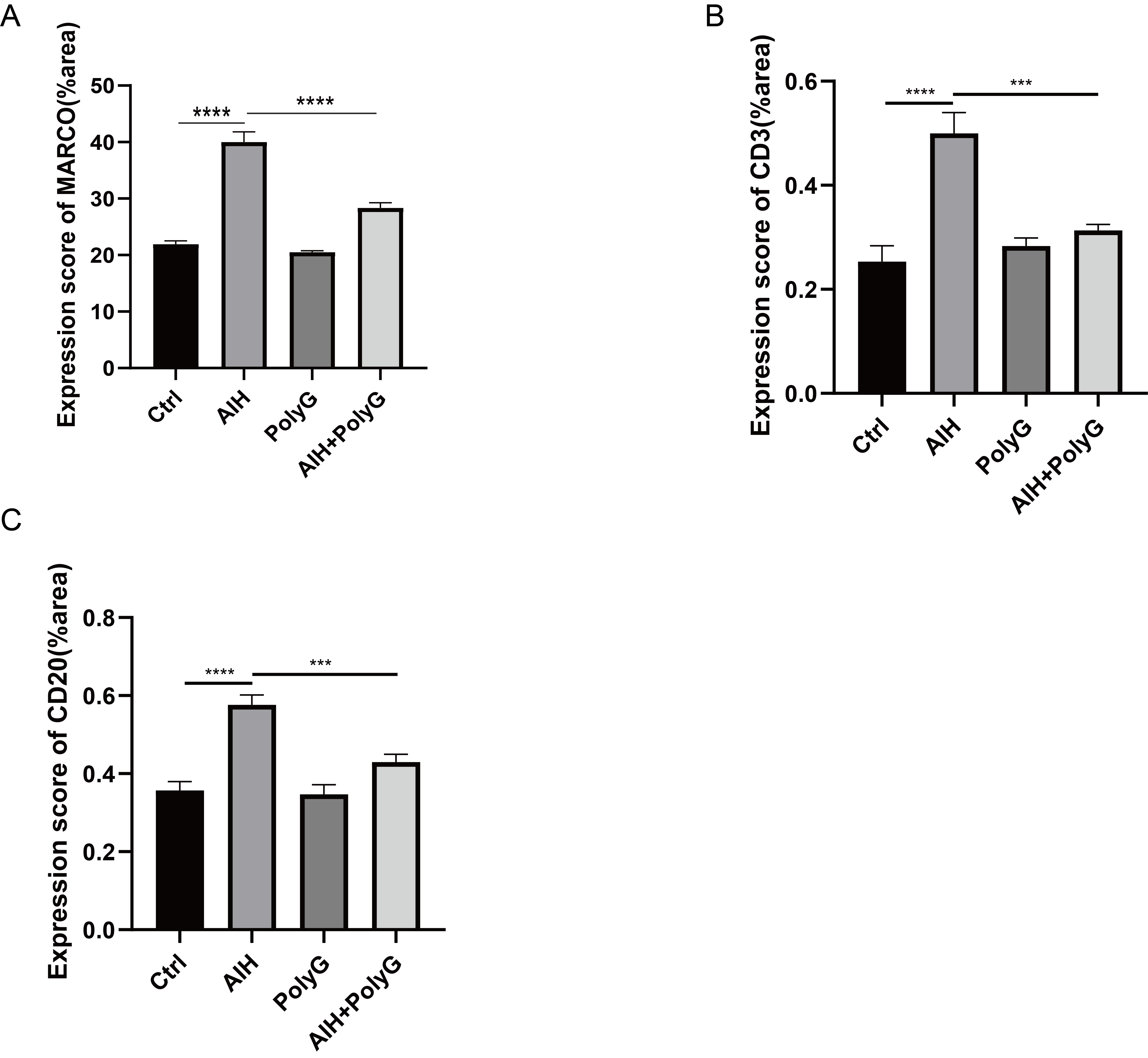

Supplement: Supplementary file 2 — Figure S2 [file JCMM-26-5690-s002.png]
