# Supplementary material for: Polyguanine alleviated autoimmune hepatitis through regulation of macrophage receptor with collagenous structure and TLR4‐TRIF‐NF‐κB signalling
Source: J Cell Mol Med. 2022 Oct 25;26(22):5690–701. doi: 10.1111/jcmm.17599 (PMC9667514; doi:10.1111/jcmm.17599)
Supplement: Supplementary file 3 — Tables S1‐S2 [file JCMM-26-5690-s003.pdf]

# Supporting Information

## Polyguanine (PolyG) alleviated autoimmune hepatitis through regulation of macrophage receptor with collagenous structure (MARCO) and TLR4-TRIF-NF-κB signaling

**Table 1 The sequences for nonspecific control siRNA and MARCO specific siRNA**

| MARCO | Sense | Antisense |
|---|---|---|
| MARCO | GGGUGACACAGGAAUUCAATT | UUGAAUUCCUGUGUCACCCTT |
| Non-specific control | UUCUCCGAACGUGUCACGUTT | ACGUGACACGUUCGGAGAATT |

**Table 2 The primer sequences of genes in real-time qPCR assay**

| Gene | Species | Forward | Reverse |
|---|---|---|---|
| MARCO | Mouse | CAGGAAACAAAGGGGACA | GGAGATCCAGCCAAACC |
| IL-1β | Mouse | CCCAAGCAATACCCAAAGAA | GCTTGTGCTCTGCTTGTGAG |
| IL-6 | Mouse | GGCAAGCCTTCCAGTTAGTCTTCC | AGAGTAAGCGTCCAGAGGTCAGC |
| TNF-α | Mouse | TTCTGTGAAAACGGAGCTG | AAAAGAGGAGGCAACAAGG |
| I-κB | Mouse | TGCCGTGACCTCAAGATGTG | CACAAGCGTGCTGTAGGTGA |
| NF-κB | Mouse | GTGCTATGTCGCTCTGGACTTTGA | ATGAAAGATGGCTGGAAGAGGGTC |
| GAPDH | Mouse | CTCTCCCTCACGCCATC | ACGCACGATTTCCCTCTC |
