# Supplementary material for: Polyguanine alleviated autoimmune hepatitis through regulation of macrophage receptor with collagenous structure and TLR4‐TRIF‐NF‐κB signalling
Source: J Cell Mol Med. 2022 Oct 25;26(22):5690–701. doi: 10.1111/jcmm.17599 (PMC9667514; doi:10.1111/jcmm.17599)
Supplement: Supplementary file 4 — Figure captions [file JCMM-26-5690-s004.docx]

Figure S1: The role of complete Freund's adjuvant in AIH. Panel A shows the levels of serum ALT and AST in each group.Panel B displays the representative pictures of H&E staining. Panel C shows histology scores (based on ishak inflammation score).Panel D represents the typical Western blot for TNF-a, Ik-B and NF-kB. GAPDH and Lamin B were used as loading control. * indicates P < 0.1,and **** indicates P < 0.0001.

Figure S2: Expression score of liver in mice. Panel A shows the MARCO expression score in liver of mice. Panel B represents the CD3 expression score in liver of mice. Panel C displays the CD20 expression score in liver of mice.
